# Lessons learned: next-generation sequencing applied to undiagnosed genetic diseases

**DOI:** 10.1172/JCI154942

**Published:** 2022-04-01

**Authors:** Bryce A. Schuler, Erica T. Nelson, Mary Koziura, Joy D. Cogan, Rizwan Hamid, John A. Phillips

**Affiliations:** 1 Division of Medical Genetics and Genomics and; 2 Department of Pediatrics, Vanderbilt University Medical Center, Nashville, Tennessee, USA.

## Abstract

Rare genetic disorders, when considered together, are relatively common. Despite advancements in genetics and genomics technologies as well as increased understanding of genomic function and dysfunction, many genetic diseases continue to be difficult to diagnose. The goal of this Review is to increase the familiarity of genetic testing strategies for non-genetics providers. As genetic testing is increasingly used in primary care, many subspecialty clinics, and various inpatient settings, it is important that non-genetics providers have a fundamental understanding of the strengths and weaknesses of various genetic testing strategies as well as develop an ability to interpret genetic testing results. We provide background on commonly used genetic testing approaches, give examples of phenotypes in which the various genetic testing approaches are used, describe types of genetic and genomic variations, cover challenges in variant identification, provide examples in which next-generation sequencing (NGS) failed to uncover the variant responsible for a disease, and discuss opportunities for continued improvement in the application of NGS clinically. As genetic testing becomes increasingly a part of all areas of medicine, familiarity with genetic testing approaches and result interpretation is vital to decrease the burden of undiagnosed disease.

## Introduction

Rare and undiagnosed diseases can have major impacts on affected individuals, and increased understanding of these diseases has led to many biological discoveries. In the United States, rare genetic diseases are defined as affecting fewer than 200,000 individuals, which corresponds to a prevalence of approximately 86 per 100,000. However, rare genetic diseases are relatively common when considered in aggregate, with an estimated population prevalence of between 3500 and 5900 per 100,000 (1).

The diagnosis of genetic diseases is being revolutionized by next-generation sequencing (NGS), which includes exome sequencing (ES) and genome sequencing (GS). NGS has accelerated molecular insights into the etiology of genetic disorders. However, while ES is considered to have high diagnostic utility, it fails to provide a diagnosis in a substantial number of cases (2–7). Better understanding of the strengths and limitations of our current molecular diagnostic approaches can help facilitate continued discovery of the molecular basis of disease as well as contribute to our knowledge of both the function and dysfunction of the human genome.

Given the rapid evolution of technologies that have made the clinical implementation of genetic testing possible, several review articles have addressed aspects of NGS related to its use diagnostically, including its use in the pediatric population (8); the bioinformatics approaches necessary for NGS data analysis (9); and laboratory-centric data generation and interpretation (10).

This Review discusses a variety of genetic testing approaches not limited to NGS, highlights the strengths and weaknesses of genetic testing strategies, and suggests mechanisms to improve clinical applications of NGS. As more primary care and subspecialty providers order genetic testing, an understanding of what these genetic testing techniques do and do not test is important to avoid drawing incorrect conclusions from genetic tests, especially avoiding the assumption that a single negative genetic test rules out genetic disease.

## Types of genetic tests

Genetic testing approaches vary based on clinical indications and have evolved over time to include NGS. In 1999, the Task Force on Genetic Testing defined a genetic test as “the analysis of human DNA, RNA, chromosomes, proteins, and certain metabolites in order to detect heritable disease-related genotypes, mutations, phenotypes or karyotypes for clinical purposes. Such purposes include predicting risk of disease, identifying carriers and establishing prenatal and clinical diagnosis or prognosis. Prenatal, newborn and carrier screening, as well as testing in high-risk families, are included” (11).

### Newborn screening.

In many countries, newborn screening (NBS) is used to rapidly identify neonates with treatable genetic conditions. NBS uses a combination of tandem mass spectrometry, gel electrophoresis, enzymatic activity assays, and gene sequencing (12). The goal of NBS is to rapidly identify neonates at high risk for prompt evaluation and treatment. Inclusion of disorders in NBS varies based on location, but typically includes disorders of amino acid metabolism (e.g., phenylketonuria, maple syrup urine disease), disorders of fatty acid oxidation (e.g., medium-chain acyl-CoA dehydrogenase deficiency), disorders of carbohydrate metabolism (e.g., galactosemia), hemoglobinopathies (e.g., sickle cell anemia), and cystic fibrosis. Importantly, NBS is a screening test, not a diagnostic test. It requires rapid assays of optimally collected blood spot samples and prompt follow-up testing to solidify a diagnosis.

### Biochemical studies.

Laboratory biochemical evaluations are used to assess for inborn errors in metabolism (IEM) because of their greater sensitivity (in some cases) and faster turnaround time compared with NGS. They include tests such as acylcarnitine profiles, plasma amino acids, urine organic acids, ammonia levels, free and total carnitine, creatine metabolites, vitamin levels, and complete metabolic profiles. They can provide biochemical evidence for an underlying IEM and, in some cases, be diagnostic without a confirmatory molecular test. As there is overlap in the techniques used by NBS and these biochemical studies, there is overlap in detectable diagnoses. Biochemical studies can be used for confirmation of NBS results and/or uncover disorders of creatine metabolism, urea cycle disorders, carnitine deficiency, and mitochondrial dysfunction. A study showed that, while NGS was able to diagnose 50% of the cases with a suspected IEM, specific biochemical profiles can provide phenotypic data that increase the likelihood of a diagnosis with NGS (13).

### Karyotype and chromosomal microarray.

**Figure 1:**
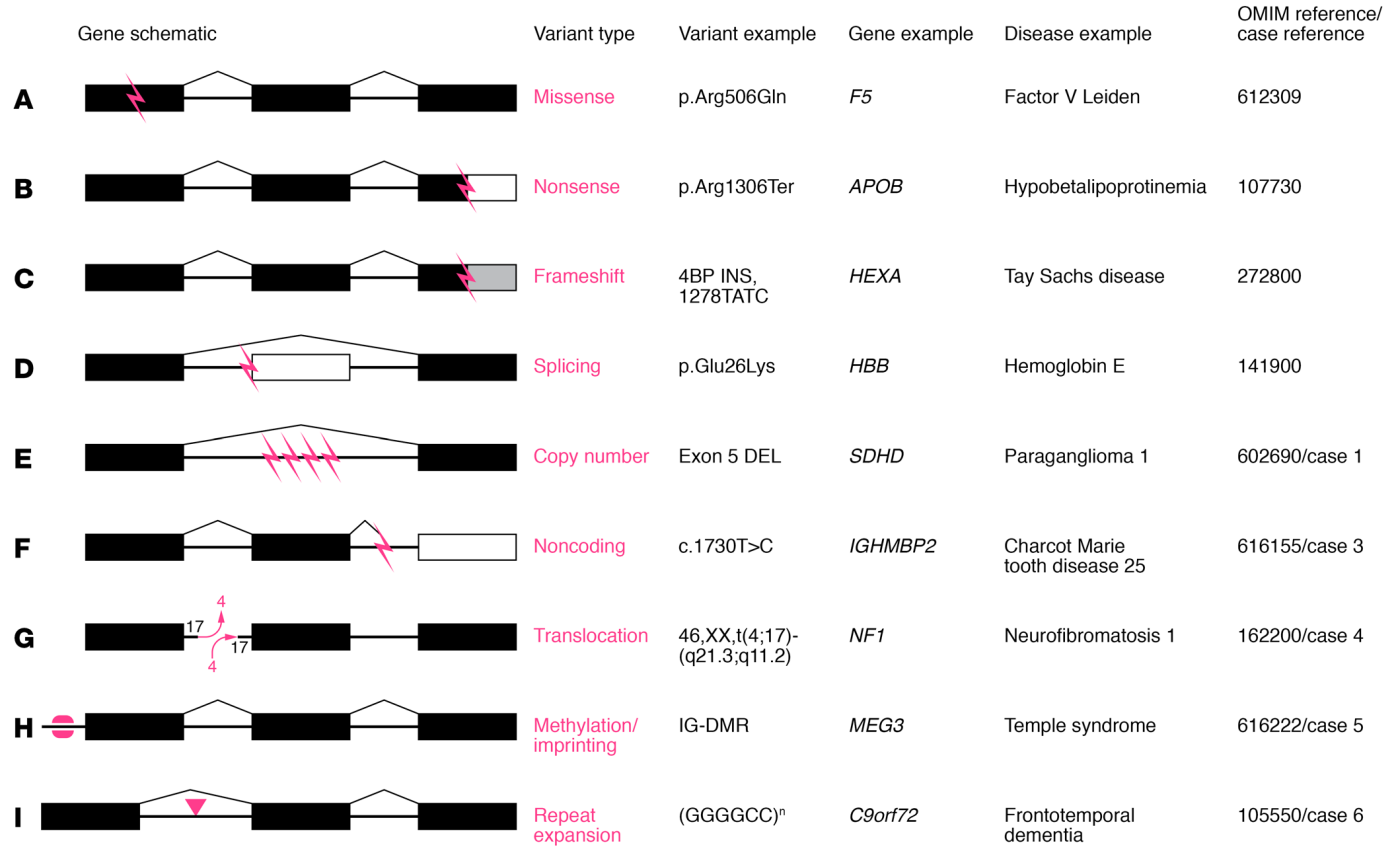
Schematic diagrams representing different types of genomic variation. By column from left to right are diagrams depicting a gene schematic with a variant, the type of variant, and a specific example of that type of variant. Those columns are followed by example genes that have been demonstrated to harbor that kind of variant, a representative disease caused by that type of variant, and the OMIM reference for that disease. The last column additionally includes references to cases in this Review, if applicable.

For decades, karyotypes were the only means to identify chromosomal abnormalities including aneuploidies (abnormal numbers of chromosomes) and large (more than 3–10 Mb) deletions or duplications (Table 1 and refs. 14, 15). With the development of chromosomal microarrays (CMAs), there has been a large shift from the use of karyotypes to the use of CMAs as a first-line genetic test in evaluating individuals with developmental delay, intellectual disability, multiple congenital anomalies, and autism spectrum disorders (15–17). While there are several different types of CMAs, all use fluorescence to genotype and count the number of alleles at millions of locations across the genome. As a product of this resolution, CMAs have increased sensitivity for deletions and duplications, with newer platforms detecting copy number variants (CNVs) as small as 30 kb (Table 1, Figure 1, and ref. 15). CMAs will still fail to detect balanced chromosomal translocations and have poor sensitivity in detecting inversions and triploidy; karyotypes are still better for the detection of translocations (Figure 1) and aneuploidies. While the ability of sequencing analysis to detect CNVs continues to improve, short-read, exon-only sequencing platforms still miss many CNVs (Table 1 and ref. 18).

### Sequencing.

The practice of clinical genetics has evolved in parallel with testing strategies and knowledge of the genome. Historically, many diagnoses were based on clinical diagnostic criteria rather than a molecular test. As more was learned about phenotypic and genotypic diversity of genetic disorders and our ability to sequence more genes in a cost- and time-efficient manner evolved, practice shifted from the use of single-gene sequencing to gene panels in which multiple genes whose dysfunction could cause overlapping phenotypes are sequenced. Eventually these gene panels incorporated NGS for disorders that were not detected by karyotype, CMA, or fluorescence in situ hybridization (FISH) analyses (Table 1). Single-gene sequencing or gene panels are often used when specific diagnoses are suspected (e.g., neurofibromatosis, Noonan syndrome, or CHARGE syndrome) or when there are phenotypes that overlap between genetic disorders (e.g., congenital heart disease, autism spectrum disorders, or skeletal dysplasias).

## Variant interpretation

Sequencing tests can identify variants that are defined as nucleotide differences from the reference sequence. Missense variants are those that produce a single amino acid change (Figure 1). Nonsense variants are those that result in premature stop codons leading to early termination of the protein sequence (Figure 1). Subsequently truncated proteins can either be dysfunctional or be subjected to nonsense-mediated decay. Splicing variants interfere with pre-mRNA processing so that the final mRNA that is translated includes inappropriate intronic sequences and/or excludes exonic sequences (Figure 1). Promoter variants can alter the affinity of RNA polymerase for the promoter site, which can affect the amount of mRNA produced. Synonymous variants change the DNA sequence at the codon level, but the amino acid encoded by the new codon does not change because of redundancy in the genetic code. The ability to detect these single-nucleotide variants differs between sequencing platforms. Exon-based sequencing should detect missense, nonsense, and synonymous variants and may or may not detect variants that affect splicing, depending on the location of the variant and how much of the intron/exon boundary is captured. ES approaches tend to miss regulatory or promoter variants. GS should capture all types of single-nucleotide variants (Table 1).

In addition to determining the effect the variant has on the expression and/or processing of the encoded mRNA and its protein product, the clinical impact of a variant can be difficult to determine; this is especially true for missense and synonymous variants. The American College of Medical Genetics and Genomics (ACMG) has provided guidelines for variant classification that weighs evidence to classify variants as pathogenic, likely pathogenic, of uncertain significance, likely benign, or benign (19).

Evidence for variant classification is gathered from published literature, bioinformatics databases, and in silico tools. Population databases, like the Genome Aggregation Database (gnomAD) (20), report variant frequencies in the general population to address the thought that pathogenic variants should appear at a population frequency corresponding to the prevalence of the genetic disease. GnomAD has data from more than 125,000 exomes and more than 15,000 genomes from individuals who are not biologically related, represent a variety of ancestries, and are presumably healthy (20). Conversely, disease databases, like ClinVar, contain variants found in patients with genetic disease. ClinVar has information on more than 500,000 variants, including phenotypic data and clinical interpretation (21, 22). Phenotypic information, whether reported in primary literature or databases, is crucial for genotype-phenotype correlation and thus variant interpretation. Finally, in silico models are used to predict the effects of a variant at the nucleotide, splicing, or amino acid level. The many different informatics tools available have varying strengths and weaknesses. These tools make predictions on pathogenicity based on evolutionary conservation at the amino acid level, deviation from known splicing motifs, effects on amino acid sequence, or how dissimilar the properties of the variant amino acid are versus the reference amino acid (20). Each of these sources of evidence has its shortcomings. For instance, there are individuals thought to be healthy who harbor pathogenic variants; databases are not necessarily peer-reviewed or updated frequently; and in silico prediction tools do not fully capture biological complexity and therefore may be inaccurate. Despite use of multiple tools, there is often insufficient evidence to determine whether a variant is disease-causing at the time of its discovery.

## Diagnostic rates using NGS

As use of ES has increased, it has been demonstrated to be superior to gene panels in diagnostic rate and, in some cases, cost (23). The overall reported diagnostic yield of ES varies based on institution, when the analysis was done, and the clinical indication for testing. The diagnostic rate has continued to increase from 25% in 2013–2014 (6, 7) to 28.8%–31.0% in 2016 (5). A study in 2017 demonstrated a diagnostic rate of 52% using singleton ES in children with multiple congenital anomalies (24). The continuing evolution in the use of NGS was demonstrated when GS was shown to have a diagnostic rate of 41% compared with 24% with other genetic testing approaches, including ES. The authors concluded that the ability of GS to identify structural variants and noncoding variants (NCVs) that were not detected by ES platforms explained this difference (Table 1 and ref. 18).

Sometimes a patient’s phenotype is caused by a combination of genetic diseases. Such presentations can confound NGS data interpretation, because the initial impulse is to assign one molecular cause to all the patient’s phenotypes. This can lead to diagnostic delays or inaccurate attribution of phenotypes to the wrong candidate variant and subsequent assignment of incorrect diagnoses to other patients with similar symptoms (25). One study found that 4.9% of patients who underwent ES had multiple molecular diagnoses (26). Correct phenotyping is key to NGS variant analyses and is vital to the detection of candidate variants (27–30).

## NGS limitations

While there are clinical scenarios in which NGS improves the diagnostic rate, there are others in which alternative approaches are still important and/or the standard of care. In the following section, we present several examples that illustrate different pitfalls that prevented NGS alone from providing a diagnosis for patients (summarized in Table 2). The following previously published examples are cases from our experiences and other similar experiences in which NGS was not the primary means of making a genetic diagnosis.

### Case 1: detecting copy number variants.

A 60-year-old woman with multiple benign neck paragangliomas, episodic hypertension with tachycardia, vocal cord paralysis, and an extensive family history of paragangliomas was tested with a hereditary pheochromocytoma and paraganglioma (HPP) NGS panel, which was negative. She and two of her family members then had GS, which detected a 2.17 kb deletion that included exon 5 of the *SDHD* gene (Table 2 and Figure 1). Subsequent high-density CMA confirmed the deletion, resulting in a diagnosis of paragangliomas 1 (31) (OMIM #168000; ref. 32). Deletions in *SDHD* account for as many as 10% of HPP-causing variants (33). While *SDHD* was included in the original panel, the testing did not include deletion and duplication (i.e., copy number variant) testing. NGS testing, including gene panels, ES, and GS, can detect some copy number variants (CNVs), but dedicated CNV testing should be considered if it was not included with the single-gene or gene-panel sequencing or if the sequencing test failed to uncover a CNV and the clinical suspicion remains high (34).

### Case 2: detecting mosaicism.

A 5-year-old boy with developmental delay, atrial septal defect, hypotonia, and skin pigmentation variation was found to have diploid/triploid mosaicism (DTM) via GS of skin biopsies. DTM occurs when some cells have three sets of chromosomes (triploid) and the remainder have the normal two copies (diploid). The patient’s GS diagnosis was confirmed by a karyotype (31). While both a karyotype and GS resulted in the same diagnosis, testing could have started with a CMA for his developmental delay based on 2010 ACMG guidelines (35). A CMA would have identified this patient’s mosaicism, which could then be confirmed by a karyotype. Retrospectively, this testing strategy could have prevented GS, which is a relatively time-consuming and expensive test in comparison with CMA or karyotype (Tables 1 and 2). It could alternatively be argued that the patient’s variation in skin pigmentation could be a manifestation of underlying mosaicism and that an NGS strategy as first-tier testing on a limited source of DNA (i.e., the skin biopsy) would be indicated, especially in the context of the recent recommendations by the ACMG (36). The ideal testing strategy is not always obvious based on patient presentation, but continued consideration of what various genetic tests can and cannot detect can increase the likelihood of a diagnosis. In cases in which the chromosomal change is too small to be identified with a karyotype, CMA or NGS would be needed to identify mosaicism (31).

### Case 3: detecting noncoding variants; using transcript analysis.

A 5-year-old girl was seen for developmental delay starting at 3 months of age. She had inversion of her feet that progressed to muscle weakness, calf atrophy, and decreased lower extremity reflexes at 18 months of age. She had normal basic and metabolic biochemical laboratory evaluations, electromyography and nerve conduction studies that were consistent with motor axonal polyneuropathy, and normal MRIs of her brain and spine. She had genetic testing for spinal muscular atrophy type 3 that was negative. ES revealed a single pathogenic variant in *IGHMBP2*, a gene associated with an autosomal recessive type of Charcot-Marie-Tooth disease (OMIM #616155; ref. 32). No other variants were reported. GS revealed a deep-intronic noncoding variant (NCV), also in *IGHMBP2* (Figure 1). Reverse transcriptase PCR revealed that this variant activated a cryptic splice site and led to a frameshift insertion that resulted in a premature termination codon, and that nonsense-mediated decay caused destruction of the *IGHMBP2* transcripts. It was necessary in this case to use GS and transcript analysis to identify and prove the functional impact of the *IGHMBP2* NCV (Tables 1 and 2 and ref. 37).

### Case 4: FISH and cosegregation studies detect translocations.

A 3-year-old girl with a history of multiple café au lait macules and cervicomedullary and retropharyngeal plexiform neurofibromas met the clinical criteria for diagnosis of neurofibromatosis type 1 (NF-1) (OMIM #162200) (32). When she was a neonate, karyotype revealed a balanced translocation between her chromosomes 4 and 17 (Figure 1). Because the *NF1* gene is on chromosome 17, NGS assessed for sequencing variants in *NF1* and multiplex ligation-dependent probe amplification (MLPA) was used to look for CNVs; both tests were negative. The patient’s mother and two brothers also had clinical diagnoses of NF-1 and shared the balanced translocation. Since NF-1 cosegregated with the balanced translocation in this family, it was suspected that the translocation breakpoint disrupted the *NF1* allele (Table 2 and Figure 1). This was confirmed using FISH analysis with custom probes for both the 5′ and 3′ regions flanking the *NF1* gene (38). Gene translocations can be missed by NGS and MLPA (Table 1). With the incorporation of multiple testing approaches including NGS, the molecular diagnostic rate for NF-1 has increased from approximately 50% to approximately 95% (38).

### Case 5: detecting methylation and imprinting variants.

A 12-year-old girl was evaluated for progressive obesity, hypotonia, recurrent fractures, and developmental delays. Genetic testing included CMA, Prader-Willi methylation, mucopolysaccharidosis biochemical testing, and *GNAS* and *WFS1* sequencing. ES was non-diagnostic but detected a pathogenic *ANO5* variant and variants of uncertain significance in *ELN*, *HIVEP2*, *COL6A3*, and *LRP5*. *ANO5* was the top candidate of interest because of the patient’s history of pathological fractures and muscle hypotonia. A second pathogenic variant, which would be expected in an autosomal recessive disorder, was not detected despite deletion/duplication studies of *ANO5*. More detailed phenotyping and a literature review suggested that she might have phenotypic manifestations of Temple syndrome (a disorder caused by abnormal methylation). Methylation analysis at the *MEG3* transcriptional start site differentially methylated region on chromosome 14q32 showed complete hypomethylation (Figure 1), which was both different from the normal heterozygous methylation pattern and consistent with a diagnosis of Temple syndrome (31) (OMIM #616222; ref. 32). ES and GS will miss epigenetic disorders, and careful phenotyping combined with a high index of clinical suspicion is required to make these diagnoses (Tables 1 and 2). Therefore, methylation studies should be considered concurrently with NGS when warranted. This case illustrates why genetic evaluations should be iterative processes that include reevaluation of the phenotypes and genotypes that are used to generate differential diagnoses.

### Case 6: detecting repeat expansions.

A kindred was identified with a strong family history of amyotrophic lateral sclerosis (ALS) and frontotemporal dementia (FTD). There were ten affected individuals in an autosomal dominant inheritance pattern. These individuals experienced symptoms including loss of empathy and apathy, impaired executive function, dysphagia, apraxia, and hyperreflexia. Neuropathological findings revealed atrophy of the frontal lobes with variable involvement of other regions, neurodegeneration, and decreased myelination. Linkage analysis implicated a region on chromosome 9p as responsible for conferring this phenotype. Despite there being only 10 genes in this region, genomic sequencing and expression analysis failed to identify the gene responsible for the ALS-FTD phenotypes (39). Subsequent studies identified that a hexanucleotide repeat in *C9orf72* was responsible for ALS-FTD (40, 41) (OMIM #105550; ref. 32). A similar case of FTD was reported in which both ES and GS were non-diagnostic, but repeat expansion (RE) testing of *C9orf72* revealed more than 44 repeats, which led to the diagnosis (31). Recent work has improved the ability to identify REs from NGS data. Initial bioinformatics approaches could detect REs from NGS data if the location of the RE of interest was already known (42). Subsequent work improves detection of REs in a way that does not rely on knowing that an RE exists by leveraging the unique sequence flanking the RE to sequence into the RE. However, these approaches that use short-read sequencing do not give insight into the length or composition of the RE (43). Leveraging multiple RE detection approaches simultaneously has improved RE detection, with some caveats. GS more reliably detects REs than does ES. A combination of genotyping and statistical approaches can increase the reliability of RE detection. Reliable determination of RE length and composition remains a problem, and current RE detection often still relies on PCR or Southern blot techniques (Tables 1 and 2, Figure 1, and ref. 44).

## Improving the diagnostic utility of NGS

We have discussed types of genomic changes missed with NGS and have provided case examples in which other approaches were required to make a diagnosis (Figure 1 and Table 1). Further advancements in NGS technologies will continue to improve diagnostic utility. For example, the ACMG released a practice guideline in 2021 recommending that ES/GS be considered a first- or second-tier test in patients with congenital anomalies, developmental delay, or intellectual disability based on clinical utility for providers and families (35). Prior to this guideline, there was variability in the diagnostic approach for these patients; first-tier testing could include CMA, fragile X testing, and biochemical studies. Single-gene or gene-panel testing could be incorporated at any point in the diagnostic workup. The new guidelines are based on evidence demonstrating utility in performing ES or GS after CMA or focused testing. Even so, testing options are influenced by medical insurance, provider preferences, parental desires, and health care system policies and practices (35).

### Short-read versus long-read sequencing.

DNA sequencing technology has changed greatly in the more than 40 years since Sanger sequencing was developed. Over this time, sequencing has transitioned from gel electrophoresis–based approaches like Sanger sequencing to shotgun sequencing to the NGS approach that uses massively parallel sequencing (45). NGS has greatly decreased the cost and increased the efficiency of DNA sequencing. These technologies are primarily “sequence by synthesis,” where complementary nucleotides are added sequentially, causing nucleotide-specific fluorescence that is read by a camera and results in sequences that are 100 to 200 bp in length (46). While this approach has revolutionized the fields of genetics and genomics, short reads can be difficult to computationally align to the reference genome, which makes resolution of complex and repetitive regions of genomes difficult and severely limits detection of structural variants (Table 1, Figure 1, and refs. 47, 48). In contrast, evolving long-read sequencing approaches generally use alternative sequence by synthesis chemistry or measurement of changes in electrical current caused by the DNA molecule as it passes through a nanopore. These reads can be 10 kb to several Mb in length (46). Further advances to increase the accuracy and decrease the cost of long-read sequencing technologies could lead to a genetic testing approach that would allow for de novo genome assembly; identify sequence variation, copy number variation, REs, and structural variants; and provide more accurate phasing of variants (Table 1, Figure 1, and refs. 46, 49, 50–53). However, obstacles to realizing the full potential of long-read sequencing include that it remains relatively expensive (6- to 12-fold more expensive by some estimates) and does not have the base-calling accuracy of short-read sequencing technology (50, 53). Data storage and scalability are also issues; large genomes present the problem of storing large amounts of data as well as demonstrate decreasing efficiency of genome assembly as genome size increases. While long-read sequencing can identify epigenetic changes, these technologies have not yet realized that full potential (54). Short-read sequencing covers a majority of the known, disease-causing structural variants, and consequently it is thought that the addition of long-read sequencing technology, in its current state, is unlikely to substantially increase diagnostic yield (50). As the sequencing technology continues to improve, the cost decreases, and the bioinformatics pipelines become more accurate and efficient, long-read sequencing may eventually contribute to an increased diagnostic yield from genetic testing (46, 50, 53, 54).

### Data sharing and cloud computing.

Another opportunity to improve our understanding of genomic function and dysfunction comes from leveraging large genomic databases that are coupled with phenotypic data. Currently, ClinVar and gnomAD are two of these most-used databases. ClinVar has partnered with a collaborative program called Clinical Genome Reference (ClinGen) to improve the curation, sharing, and archiving of genomic variation data as well as their clinical interpretation or relevance. ClinGen curates data for ClinVar from other databases and structures data submissions into proper format and nomenclature. The program also developed a system to define the review level of submissions. To aid in reviewing submissions, ClinGen develops expert teams in various clinical realms to validate variant pathogenicity and gene-disease relationships (55).

An example of the power of using large databases comes from Brokamp et al., who reported a patient in whom they identified a de novo frameshift variant that had not been observed previously (56). There were no matches in the available matching tools (GeneMatcher, MyGene2, Matchmaker Exchange; refs. 57–59) or in ClinVar and gnomAD. However, by utilizing their in-house database of more than 3 million individuals’ electronic health records, many of which had accompanying genomic data (i.e., BioVU; ref. 60), they found two other individuals with de novo variants in the same gene and overlapping phenotypes. By identifying multiple, unrelated individuals with variants in the same gene and with very similar phenotypes, they were able to change the designation of the variant from one of uncertain significance to pathogenic and discover a new genetic disorder (56).

The case above illustrates the potential of leveraging large data to identify other exceptionally rare cases to make diagnoses. NGS has rapidly increased the amount of available genomic data, which presents both opportunities and difficulties associated with working with petabytes (1 petabyte = 1 million gigabytes) of data to solve cases. Unfortunately, the infrastructure necessary to utilize a data set of this size is prohibitive to most clinicians and independent laboratories. Cloud computing is a system in which resources are rented to mitigate the need to establish both the hardware and software necessary for data analysis of this magnitude (61). Addressing the data sharing protocols and patient privacy concerns that come with cloud computing will be necessary to be able to utilize these platforms to their full potential.

The All of Us Research Program is an example of using cloud computing to facilitate the application of genomics in health care. This program plans to provide a resource of genomic and phenotypic data of at least 1 million people, most of whom are from backgrounds underrepresented in biomedical research. The goal of the All of Us Research Program is to create a resource of health questionnaires, electronic health record data, physical measurements, and both digital data and biospecimens for a variety of applications including characterizing natural histories of diseases, identifying disease risk factors, and revealing new biomarkers. The design of the program should mitigate the small sample sizes and lack of diversity in many genomic data sets that limit medical discovery (62). While the intention is not directly for the diagnosis of rare and/or undiagnosed disease, study participants will have the option of learning about pharmacogenomic findings as well as actionable, highly penetrant, disease-causing variants (62).

### Sequencing critically ill pediatrics patients.

Much of this Review has discussed the application of genetic testing to improve the diagnostic rate in patients for whom there is a concern regarding an underlying genetic disorder. However, critically ill patients, who may not yet present the classic signs or symptoms of a rare and unfamiliar genetic disorder, present another opportunity for the application of NGS to detect an undiagnosed genetic disease. Many severe genetic conditions present in the neonatal period or in early childhood, but the onset of characteristic signs and symptoms is delayed because they are age dependent. Multiple studies of the utility of NGS in critically ill neonates have shown increased diagnostic rates, decreased costs associated with hospitalization, changes in management, and increased patient and family satisfaction. Studies that obtained NGS on critically ill pediatric patients with concerns regarding an underlying genetic disorder yielded diagnostic rates of 21% to 58% depending on patient selection, year the study was conducted, and NGS methodology (63–72). These studies defined clinical utility as changes in medical or surgical management, testing family members for related genotypes or phenotypes, informing recurrence risk, suggesting a potential pharmaceutical, and/or involving palliative care. They reported that, in 21%–83% of cases, NGS led to a change in management regardless of whether a diagnosis was made (63–68, 70–72). For those patients in whom there was a suspicion of a genetic disease, studies showed that either ES or GS yielded an increased diagnostic rate when compared with gene panel alone or standard genetic testing approaches: 58% with trio-based ES versus 12.5%–25% with gene panels (64), 57% with ES versus 13.75% with standard approaches (66), and 57% with GS versus 9% with standard approaches (70). The Newborn Sequencing in Genomic Medicine and Public Health randomized controlled trial 1 (NSIGHT1) was a program that tested the hypothesis that rapid GS “increased the proportion of [critically ill] infants receiving a genetic diagnosis within 28 days.” NSIGHT1 was terminated early because GS demonstrated an obvious clinical benefit compared with the standard approaches (73). Finally, one study showed that, in patients with a low suspicion of an underlying genetic disorder, NGS achieved a genetic diagnosis in 53% of their cases (71).

## Discussion

The diagnostic odyssey of patients with undiagnosed genetic diseases can be shortened with NGS and an iterative approach that incorporates clinicians, bioinformaticians, and research teams (Table 2). From such collaboration, focused sequencing and sequence reanalysis may result in a higher diagnostic yield without additional costly tests. However, we suggest that when specific gene or gene-panel testing approaches do not provide a clear answer, GS should be increasingly considered before ES, if available, as GS provides increased coverage and diagnostic yield over ES. We believe that combining robust clinical phenotyping and ES/GS analysis can further improve diagnostic rates, thus informing natural history, management guidelines, recurrence risks for family members, and access to clinical trials.

The Undiagnosed Diseases Network (UDN) demonstrates the value of an iterative and team-based approach to genetic diagnoses. The UDN was established in 2014 to provide a multidisciplinary approach that applied innovative technologies to evaluate and diagnose undiagnosed disease. It is a multicenter program funded by the National Institutes of Health and was originally composed of seven clinical sites, two sequencing cores, and a coordinating center. A central biorepository, a metabolomics core, a model organism screening center, and additional clinical sites have been added (74). Even though one-third of those accepted to the UDN had already undergone ES, the UDN achieved a clinical diagnosis rate of 35%, a specific therapy was recommended for 21%, and 31 new syndromes were identified by 2018 (74). Among those who received a diagnosis, the average cost of their care before UDN acceptance was $305,428, compared with the average cost of their UDN evaluation of $18,903 (6% of the total cost). These cost estimates suggest that the UDN approach has the potential to end expensive medical diagnostic odysseys, and are consistent with analyses of the cost-effectiveness of ES (24, 74–76).

Most genetic testing approaches currently focus on sequencing coding regions (exomes) because most of our understanding of variant pathogenicity is centered on a variant’s effect on protein structure and function. This approach will inherently miss pathogenic variation (Figure 1 and Table 1). Short-read sequencing makes the identification of structural variants difficult. Long-read sequencing platforms should improve our ability to detect structural variants that are too large to be detected with short-read sequencing technology but too small to be detected by CMA (77, 78). Deep-intronic and regulatory variants are also missed with ES strategies (Table 1 and Figure 1). GS and/or RNA sequencing can help identify variants and clarify the effects of NCVs on transcript splicing and gene expression (Table 1 and ref. 79).

Interpretation of genomic test results continues to be complicated. Variants of uncertain significance often require multiple clinical visits and reanalysis, which can be time- and resource-consuming (79). An increase of genomic data with more efficient access is needed to advance our knowledge of how genomic variation leads to disease. However, sharing genetic information to diagnose disease introduces concerns about patient privacy, compatibility across platforms, and expense. Improvements are needed to improve data sharing so that our understanding of the clinical impact of genomic variation can continue to advance (Table 2 and ref. 80).

Consideration must also be given to genetic testing approaches within societal and cultural contexts. While the cost of genetic testing has decreased over time, there continue to be concerns about direct cost to patients. Evidence shows that while the out-of-pocket cost to patients has decreased and insurance coverage has improved, there are still barriers to testing access (81). Perceptions of genetic testing vary across cultural boundaries, and access to genetic testing in underserved populations remains a problem. Efforts are being made to increase access for those populations, but more work is needed to ensure that this is done in a socially conscious and culturally sensitive way (82). Genomic medicine programs are being developed across the world to foster the inclusion of all populations in the application of genomics while simultaneously respectfully collaborating across borders (83).

As genetic testing becomes more commonplace, providers in all areas of medicine will need to understand the limitations of each testing strategy to ensure that their patients receive an appropriate evaluation (Table 1). We hope to dispel a common misconception that all genetic diagnoses have been effectively excluded from consideration if a single genetic test is non-diagnostic. Expansion of genetics and genomics education will be vitally important, as there is an increasing appreciation of the pervasiveness of clinically significant genetic disease in all fields of medicine. However, while education is likely beneficial, effecting change in genetic testing practices seems to be complex and multifactorial (84).

Incredible strides have been made in the application of genomics in the clinical setting. Despite these technological advances, opportunities for continued improvement remain. This Review attempts to provide an overview of tests done in the evaluation of undiagnosed disease, including their common uses, strengths and weaknesses of each approach, and how NGS can be incorporated. Continued advancements in sequencing technology, safe and efficient data sharing, efficient and accurate variant interpretation, and reliable identification of all types of genomic variation should progressively improve the clinical utility of NGS.

## Figures and Tables

**Table 2:**
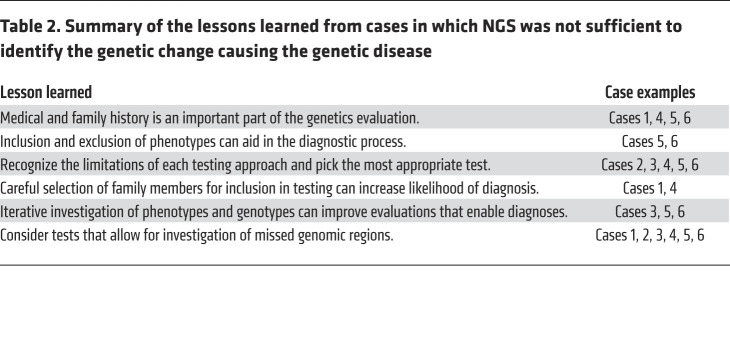
Summary of the lessons learned from cases in which NGS was not sufficient to identify the genetic change causing the genetic disease

**Table 1:**
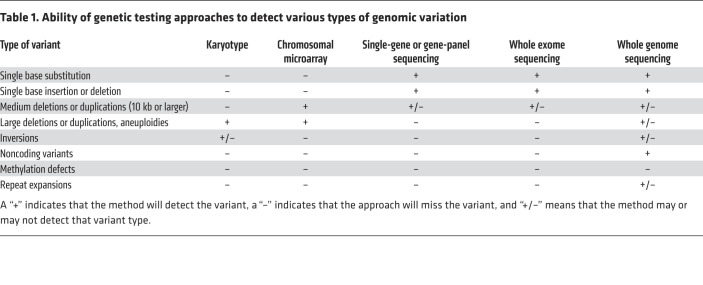
Ability of genetic testing approaches to detect various types of genomic variation

## Version 1 04/01/2022

Electronic publication
